# Public attitudes towards people who stutter in South Egypt

**DOI:** 10.1371/journal.pone.0245673

**Published:** 2021-02-04

**Authors:** Ahmed Arafa, Shaimaa Senosy, Haytham A. Sheerah, Kenneth St. Louis

**Affiliations:** 1 Department of Public Health, Faculty of Medicine, Beni-Suef University, Beni-Suef, Egypt; 2 Department of Social Medicine, Graduate School of Medicine, Osaka University, Osaka, Japan; 3 Department of Communication Sciences and Disorders, West Virginia University, Morgantown, West Virginia, United States of America; University of Haifa, ISRAEL

## Abstract

**Purpose:**

Stuttering is a multifactorial speech disorder with significant social and psychological consequences. There is a lack of knowledge about public attitudes towards people who stutter (PWS) and the factors that can determine such attitudes in underprivileged communities. This study aimed to assess the public attitudes in South Egypt towards PWS and compare our results with those stored in a reference database representing 180 different samples.

**Methods:**

A multi-stage random sampling approach was used to recruit 650 people from Beni-Suef City in South Egypt. All participants were interviewed using the Arabic version of the *Public Opinion Survey of Human Attributes-Stuttering (POSHA-S)* after getting their informed consent. This instrument assesses people’s Beliefs and Self Reactions towards PWS in addition to their sociodemographic characteristics.

**Results:**

The Beliefs and Self Reactions subscores in addition to the Overall Stuttering Score of the Egyptian sample were remarkably lower than the median values of the reference database (12 versus 34), (-4 versus 2), and (4 versus 18), respectively. TV, radio, and films were the main sources of knowledge about stuttering. Egyptian participants who reported average to high income were more likely to have a positive attitude (≥50% of Overall Stuttering Score) towards PWS than their counterparts with low income (Odds Ratio = 1.57, 95% Confidence Interval: 1.08–2.28).

**Conclusion:**

People in South Egypt showed a less positive attitude towards PWS compared with other populations worldwide. Further studies should focus on changing the public attitudes towards PWS through awareness programs that consider the cultural perspectives of the society.

## Introduction

Stuttering is a multifactorial speech disorder that can be classified according to etiology (neurogenic and psychogenic), symptomatology (tonic and clonic disfluency), and the presence of family history. This disorder affects up to 8% of people during their life span and has potential social and psychological consequences, however, its recovery rates exceed 50% [1]. Negative attitudes towards people who stutter (PWS) have been documented among different communities, ages, and professions [2–9]. PWS are perceived as fearful, worthless, timid, nervous, and introverted [10, 11]. They are vulnerable to discrimination, occupational obstacles, and bullying which lead to stigmatization and poor quality of life [12–16].

Many cultural, educational, and socioeconomic perspectives are associated with people’s attitudes towards PWS [8, 17]. Although these attitudes have been heavily studied in high and middle-income communities [2–7], data from underprivileged communities with low socioeconomic indices is sparse. Beni-Suef governorate in South Egypt is one of these communities. According to the 2017 census, almost 3.15 million people live in the governorate. Even though 47% of residents are younger than 20 years, 812,000 people older than 10 years cannot read or write and 960,000 people have never attended schools. More than 14,000 households have no access to drinkable water, 2,000 households have no electricity, only a third of households are connected to the public network of sewage disposal, the crowding index (inhabitants/room) is 1.3, and less than half of residents are covered with health insurance. Based on the same census, 0.7% of people older than five years have communication disorders (understanding or being understood) [18]. Geographically, Beni-Suef governorate is divided into seven cities and Beni-Suef City is the capital of Beni-Suef governorate which is located 120 Kilometres south of Cairo. Beni-Suef City is constituted of an urban metropolitan surrounded by rural villages, and up to 40% of its 560,000 residents live in the rural side [19].

In addition to the lack of knowledge about underprivileged communities, particularly those in the Middle East and North Africa, many of the published studies used convenience sampling methods that undermined their generalizability. Moreover, the sociodemographic factors that could determine people’s attitudes towards PWS, especially in underprivileged communities, need to be further addressed [2–9]. Two previous studies from Egypt; one from South Egypt and the other one from Cairo, assessed the attitudes towards stuttering, however, both studies were conducted on limited numbers of parents and family members of stuttering children and assessed their attitudes towards children who stutter [8, 9].

Herein, we conducted this study to assess the public attitudes of a large sample of Beni-Suef City's residents towards PWS using a multi-stage random sampling approach before comparing our results with those stored in a reference database representing 180 samples. We also sought to detect the sociodemographic factors that affect public attitudes towards PWS.

## Methods

### Study design

This cross-sectional, population-based study was conducted in Beni-Suef City during the period between April and July 2018. Our eligibility criteria included Egyptian citizens living in Beni-Suef City and aged 18 years or more. The Research Ethics Committee of the Faculty of Medicine, Beni-Suef University approved the protocol and the required institutional approvals were obtained. The study was conducted in full accordance with the guidelines for the Declaration of Helsinki.

### Sampling

The least sample size was calculated using the Epi-Info version 7 Stat Calc, [Center for Disease Control (CDC), World Health Organizations (WHO)] using the following criteria: population size of 999,999, expected prevalence of positive attitudes towards PWS of 50%, confidence level of 95%, and a margin of error of 5%. However, we more than doubled the required sample size to enhance the statistical power of the study.

To select the participants, we adopted a multi-stage random sampling approach. First, we classified the urban metropolitan of Beni-Suef City into low, middle, and high socioeconomic layers according to the prices of houses in these areas. Then, out of each layer, one district was selected randomly by card draw, and from each district, 100 households were selected using a random start. Additionally, the rural villages surrounding the urban metropolitan were stratified geographically into three locations (North, West, and South), and one rural village was chosen randomly by card draw from each location. Later, the three selected villages were clustered into two areas representing both sides of the bypassing water channel. For both urban and rural areas, householders were contacted to participate in the study.

To avoid participation bias that could be serious in the case of distributing self-administered questionnaires in a community with a high illiteracy rate, we decided to administer the questionnaire orally. Accordingly, a team of local medical students supervised by the second author of this study visited the selected households and interviewed the subjects. Uninhabited households and households belonging to other nationalities were excluded. Householders were briefed about the steps and aims of the study before inviting them to sign their informed consent and participate. No incentives were offered or given for participation. Overall, a total of 650 subjects agreed to participate in the study out of the 900 people who were invited resulting in a response rate of 72%. The main reasons for refusal to participate were being not interested or not having enough time.

### Data collection

All participants were interviewed about their attitudes towards PWS using the Arabic version of the *Public Opinion Survey of Human Attributes-Stuttering* (*POSHA-S*). The instrument was developed by *St*. *Louis* [20, 21] then translated to Arabic by *Al-Khaledi et al* [2] and *Abdalla and St*. *Louis* [3]. *POSHA-S* is comprised of three sections; section I includes sociodemographic questions such as age, sex, education, social status, income, and religion; section II includes a general comparison between stuttering and four anchor attributes (left-handedness, mental illness, obesity, and intelligence); and section III explores the Beliefs and Self Reactions to stuttering or PWS using scalar questions. The Beliefs subscore is composed of four components that measure people’s traits/personality, potential, causes, and sources of help while the Self Reaction component measures four components that address accommodation/helping, social distance/sympathy, knowledge and experience, and knowledge source. Each component is composed of at least three related items. The scores of these items, components, and subscores are transferred to a scale from -100 to +100 where higher scores refer to more positive attitudes. The Overall Stuttering Score is estimated by calculating the mean of the two components; Beliefs and Self Reactions. The *POSHA-S* has been studied for its reliability, construct and concurrent validity, internal consistency, translatability to other languages, sensitivity to differences between convenience and random sampling, and mode-equivalence between paper and online administration methods [2, 5–7, 22–24].

### Statistical analyses

Data were analyzed using the software, Statistical Package for Social Science (SPSS Inc. Released 2013. IBM SPSS Statistics for Windows, Version 22.0. Armonk, NY: IBM Corp). Frequency distributions as the percentage and descriptive statistics were calculated and our results were compared to the reference *POSHA-S* database of 14,063 subjects from 180 different samples. A radial graph representing a visual display of the scores of the participants in comparison to the lowest and highest sample means, and the median of the *POSHA-S* database was drawn. More detailed line graphs showing the subscores of the two main components; Beliefs and Self Reactions were analyzed as well. To assess the sociodemographic factors that can be associated with people’s attitudes towards PWS, multivariable logistic regression analysis was conducted and odds ratios (OR) with corresponding confidence intervals (CI) were computed. Subjects with *POSHA-S* Overall Stuttering Score ≥50% were considered of positive attitude while those <50% were considered of negative attitude. The following variables were included in the regression model: sex (male versus female), age (≤30 versus >30 years), religion (Muslim versus Christian), education (≤10 versus >10 years), income (moderate to high versus low), marriage (yes versus no), having children (yes versus no), and language (monolingual versus multilingual).

## Results

The mean age of the Egyptian participants was close to the database sample median (34.2 versus 36.9 years). More than half of the participants (56.3%) were females compared to 67% of the reference database and most of the subjects were married (82.5%) and had children (72.9%). Students represented 10% of the Egyptian sample while 62.2% were workers. More than 20% of the Egyptian participants identified themselves as intelligent, 5.2% obese, and only 0.3% considered themselves stutterers. Compared with the reference database, the self-ratings for health, abilities, and income were lower among the Egyptian participants (Table 1).

**Table 1:** Characteristics of the participating residents of Beni-Suef City in comparison to *POSHA-S* database from 180 samples.

Characteristics		Egypt	Reference database
Number		650	14,063
Age in years (Mean)		34.24	36.75
Total education years (Mean)		14.84	14.68
Sex (%)	Male	43.69	33.05
	Female	56.31	66.95
Religion (%)	Muslim	98.46	----
	Christian	1.54	----
Married (%)		82.46	53.80
Parent (%)		72.92	49.00
Student (%)		10.00	11.58
Working (%)		62.18	65.72
Not working (%)		27.08	3.09
Retired (%)		0.91	0.00
Income compared to family (-100 to +100)		3.06	8.54
Income compared to others (-100 to +100)		2.78	0.00
Multilingual		38.46	53.88
Self-identification (%)			
Stuttering		0.31	0.00
Left-handed		4.62	7.75
Obese		5.23	6.03
Mentally ill		0.00	0.73
Intelligent		20.77	27.82
Health and abilities (-100 to +100)		37	54
Physical health		18	43
Mental health		41	56
Ability to learn		33	57
Ability to speak		53	62
Impressions about (-100 to +100)			
Obese		-30	-18
Left-handed		-3	23
Mentally ill		-23	-7
Intelligent		65	57

**Fig 1:**
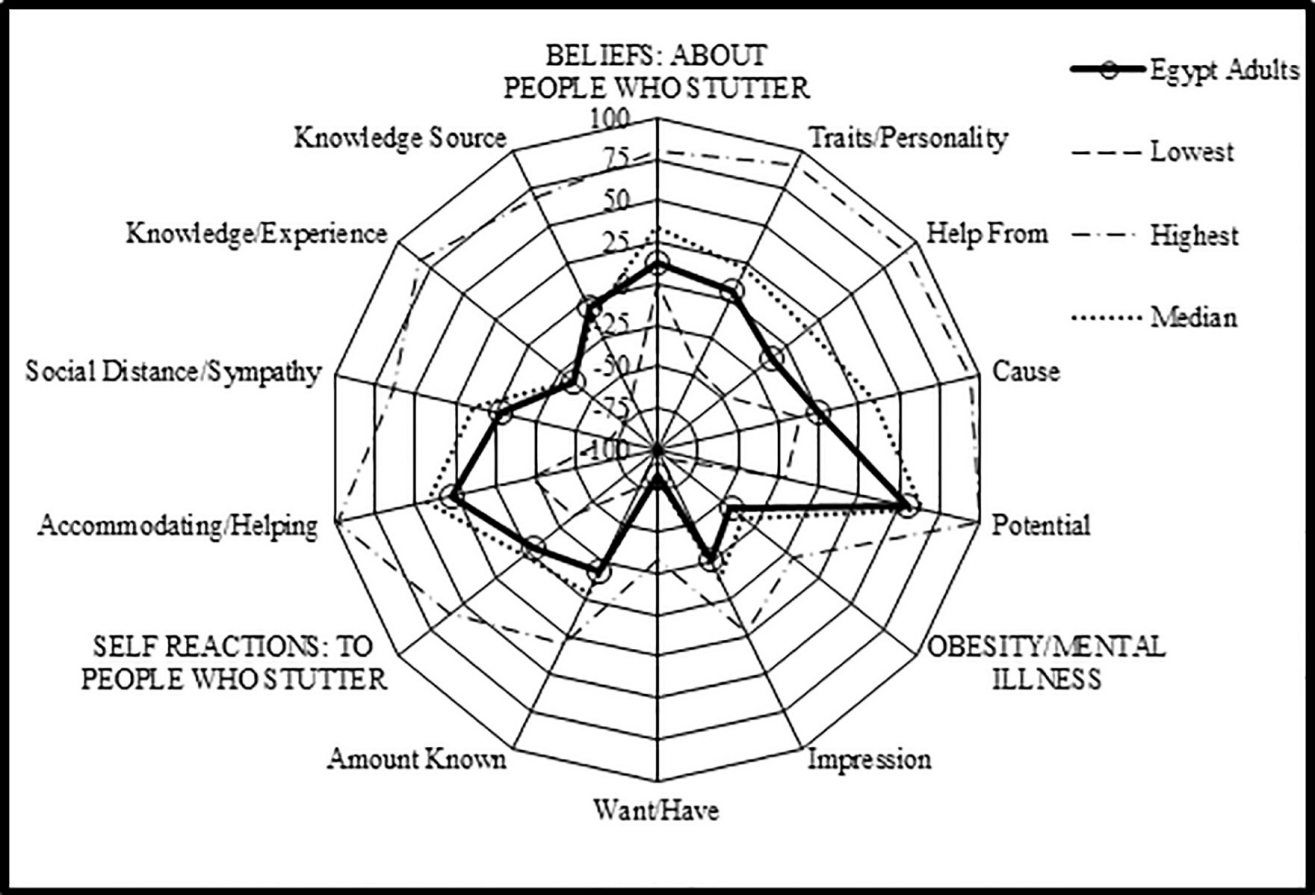
Summary of *POSHA-S* components, subscores, and overall stuttering scores of the participating residents of Beni-Suef City.

**Fig 2:**
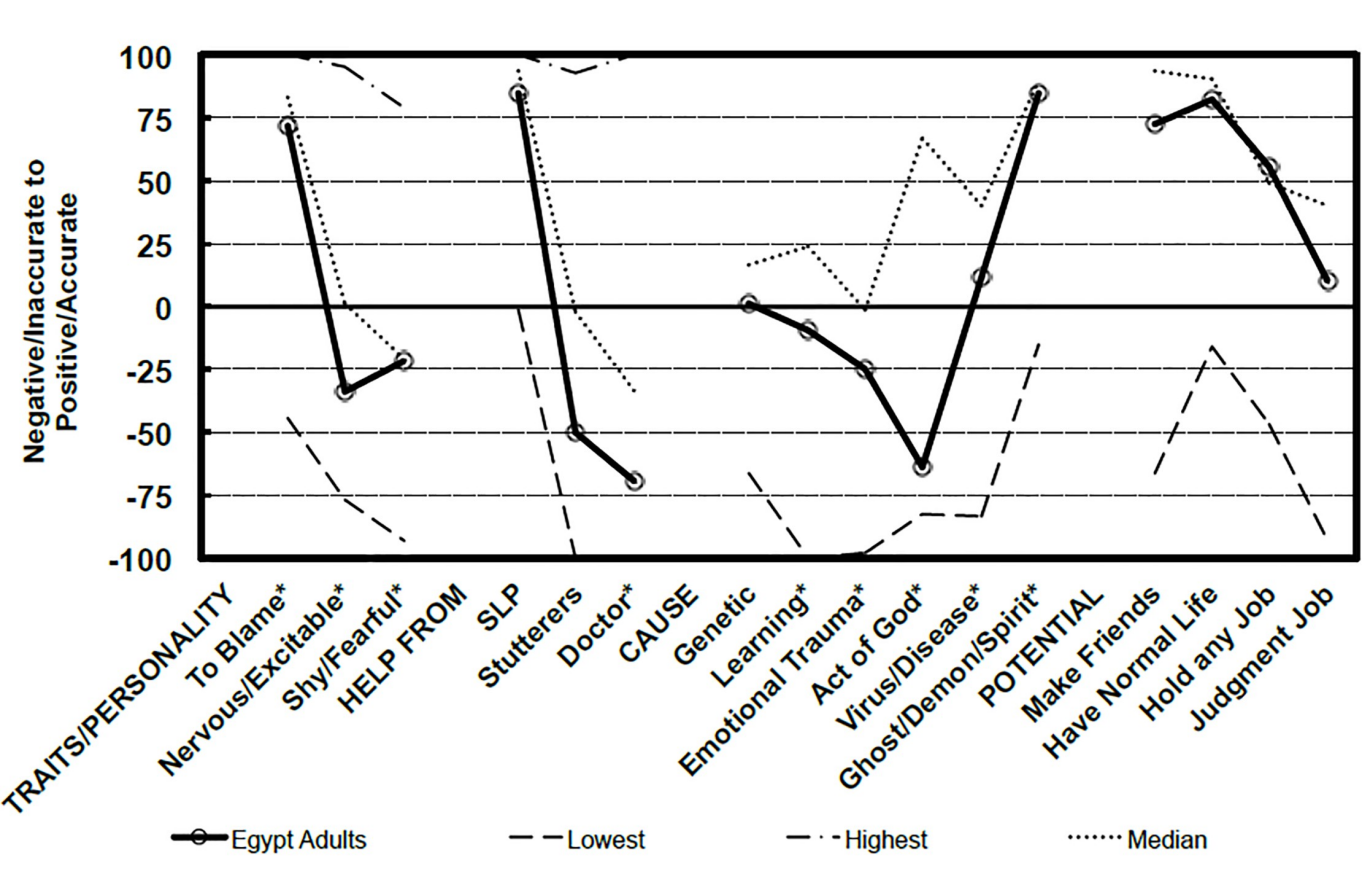
Beliefs of the participating residents of Beni-Suef City in comparison to *POSHA-S* database from 180 samples.

**Fig 3:**
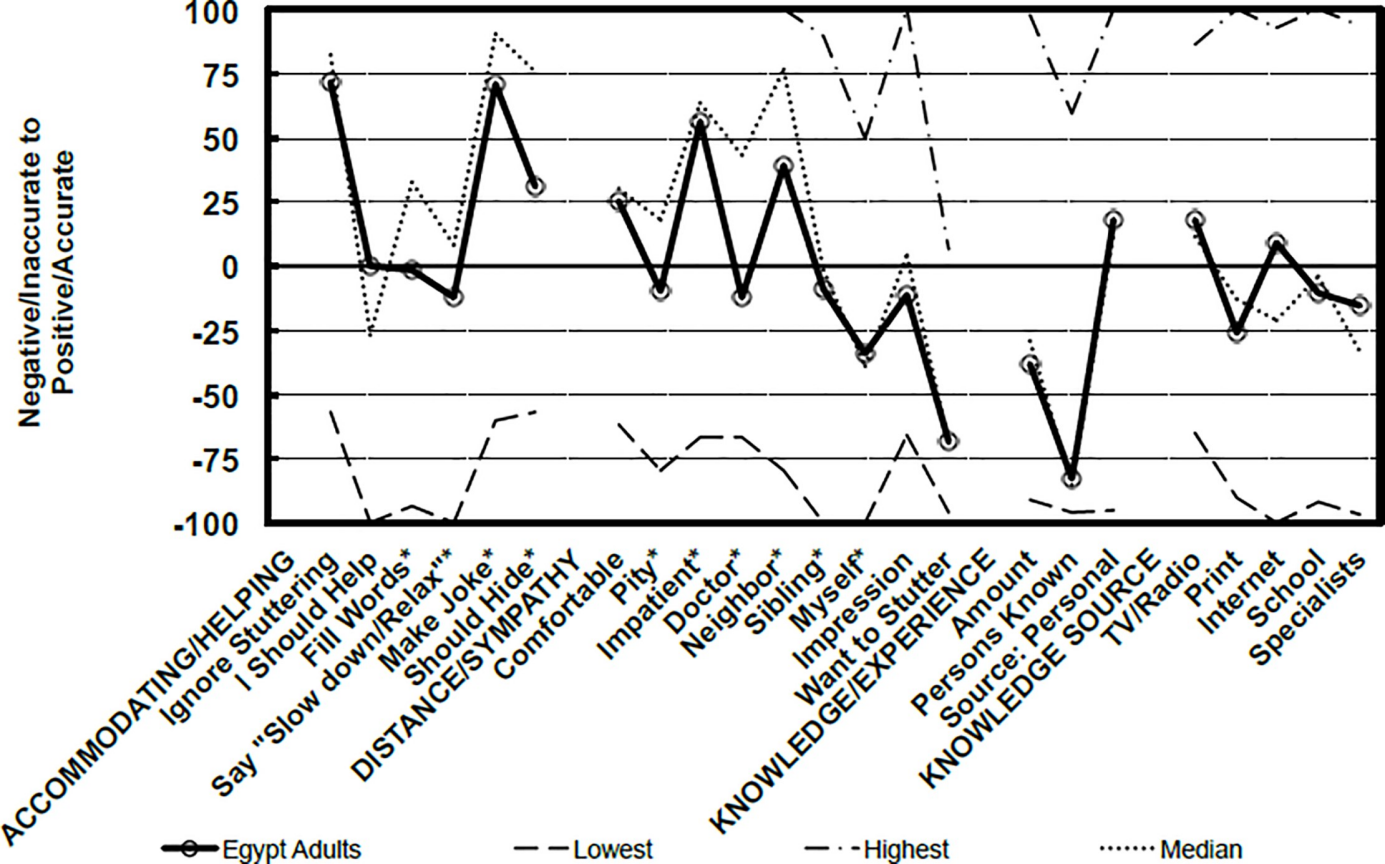
Self reactions of the participating residents of Beni-Suef City in comparison to *POSHA-S* database from 180 samples.

The *POSHA-S* Overall Stuttering Score was considerably lower among the Egyptian sample than the reference database; 4 versus 18. Compared with the 180 samples therein, only one of the means of the Egyptian sample was in the highest (most positive) quartile, while 37% were in the lowest quartile and 63% were in the middle two quartiles (interquartile range). In terms of details, the Beliefs subscore was less positive among the Egyptian sample than the reference database (12 versus 34) and for all four of its components: traits/personality (5 versus 21), potentials (55 versus 65), causes (0 versus 34), and source of help (-11 versus 16). The Self Reactions subscore of the subjects was -2 versus 4 in the reference database but lower for two of its four components: accommodation/helping (27 versus 43), social distance/sympathy (-3 versus 14), knowledge and experience (-34 for both), and knowledge source (-5 versus -11). Regarding knowledge source components, TV, radio, and films were the main sources of knowledge about stuttering while healthcare providers and magazines came last (Table 2; Figs 1–3).

**Table 2:** *POSHA-S* items, components, and subscores of the participating residents of Beni-Suef City in comparison to *POSHA-S* database from 180 samples.

*POSHA-S* variables	Egypt	Reference database
*POSHA-S* Overall Stuttering Score (-100 to +100)	4	18
Beliefs about PWS (-100 to +100)	12	34
Traits	5	21
Nervous or excitable	-34	1
Shy or fearful	-22	-21
Have themselves to blame	72	83
Potentials	55	65
Should have jobs requiring judgment	10	40
Can make friends	72	94
Can lead normal lives	82	91
Can do any job they want	55	49
Causes	0	34
Genetic inheritance	1	17
Ghosts	85	89
Frightening event	-24	-1
God’s will	-64	67
Learning or habits	-10	24
Virus or disease	12	40
PWS can be helped by	-11	16
Other PWS	-50	-2
Therapist	85	94
Doctors	-70	-34
Self Reactions to PWS (-100 to +100)	-4	2
Accommodation and helping	27	43
Try to act like the person was talking normally	71	82
Make a joke about stuttering	71	91
Fill in the person’s words	-2	33
Should try to hide their stuttering	31	76
Tell the person to “slow down” or “relax”	-12	8
Social distance and sympathy	-3	14
Feel comfortable or relaxed	25	30
Feel impatient	57	64
Feel pity	-10	18
Concern about doctor stuttering	-13	43
Concern about neighbor stuttering	39	77
Concern about brother or sister stuttering	-9	-2
Concern about me stuttering	-34	-39
Impression about stuttering	-11	4
Want to be stuttering	-68	-69
Knowledge and experience	-34	-34
Known amount about stuttering	-38	-30
Known PWS	-83	-86
Personal experiences (me, family, and friends)	18	12
Knowledge source	-5	-11
Television, radio, films	18	12
Magazines, newspapers, books	-26	-13
Internet	9	-21
School	-10	-4
Doctors, nurses, specialists	-16	-33
Obesity/Mental illness (-100 to +100)	-43	-34
Overall impression	-27	-13
Want to be	-84	-83
Amount known about	-19	-4

In the multivariable logistic regression model, people who reported average to high income were more likely to have positive attitudes towards PWS than their counterparts with low income (OR = 1.57, 95% CI: 1.08–2.28) while monolingual people and people having children were less likely to have positive attitudes compared with the multilingual ones and those with no children; (OR = 0.66, 95% CI: 0.47–0.92) and (OR = 0.53, 95%: 0.31–0.93), respectively (Table 3).

**Table 3:** Sociodemographic associations with the positive attitudes towards people who stutter in Beni-Suef City sample.

Characteristics		Overall number	Positive attitude %	Adjusted OR (95% CI)	P-value
Sex	Male	284	37.68	0.79 (0.57–1.09)	0.15
	Female	366	43.17	1	
Age (years)	≤30	242	41.32	0.99 (0.66–1.47)	0.94
	>30	408	40.44	1	
Religion	Muslim	640	40.94	1.87 (0.46–7.54)	0.38
	Christian	10	30.00	1	
Education (years)	>10	637	40.97	1.21 (0.36–4.01)	0.76
	≤10	13	30.77	1	
Income	Average to high	164	50.00	1.57 (1.08–2.28)	0.02
	Low	486	37.65	1	
Social status	Married	536	40.49	1.78 (0.93–3.42)	0.08
	Others	114	42.11	1	
Having children	Yes	474	38.61	0.53 (0.31–0.93)	0.03
	No	176	46.59	1	
Languages	Monolingual	400	36.00	0.66 (0.47–0.92)	0.01
	Multilingual	250	48.40	1	

Adjusted for sex, age, religion, education, income, social status, having children, and languages.

## Discussion

The current study showed that the *POSHA-S* Overall Stuttering Score among the Egyptian participants was 14 units, on the -100 to +100 scale, less than the median score of the reference database that represented 180 different samples. This indicates that people in South Egypt had a substantially less positive attitude towards PWS compared with other populations.

Several studies support the notion that cultural aspects have an impact on public attitudes towards PWS. *St*. *Louis and colleagues* compared *POSHA-S* scores between under and postgraduate students from Poland and the USA [25]. The authors observed that the Polish students had significantly a less positive attitude towards PWS than the American students. Still, the Beliefs and Self Reactions subscores of the under and postgraduate Polish and American students were higher than those of our sample; the *POSHA-S* Overall Stuttering Scores ranged between 14 and 31 among the Polish students and between 24 and 43 among the American students [25]. Conversely, a large study conducted on 1111 European adults recruited by convenience sampling in several different investigations showed that public stuttering attitudes in Germany (15), Bosnia (23), and Ireland/England (23) towards PWS were close to the archived database by then (17), while the attitudes from Sweden and Norway (34) were more positive than the reference database, and the attitudes from Italy (-3) were less positive. Yet, the study did not reveal significant differences among different regions from the same country. The overall differences were hypothesized to be due to cultural or citizenship-related variations across the countries [26].

One study from Poland conducted on 268 subjects representing different geographic settings revealed that the Beliefs and Self Reactions subscores and Overall Stuttering Score were 19, 8, and 14 units higher than our study, respectively [6]. A similar study on 311 adults from Portugal, but one that represented a probability sample of the entire country, showed that Beliefs, Self Reactions, and Overall Stuttering Scores were, respectively, 22, 7, and 15 units higher than ours [27]. Although the Beliefs subscores in both studies were remarkably greater than the Egyptian sample, smaller differences could be noticed regarding the Self Reactions subscore. In contrast, another study conducted on 350 adults from Hong Kong and China showed an Overall Stuttering Score of 2 to 4 units more positive than our study, with 9 to 15 units more positive for Beliefs but 6 to 7 units less positive for Self Reactions [28]. Putting the results of the Portuguese and Chinese studies side by side with our results raises the possibility that Beliefs and Self Reactions components may not be closely related in certain societies.

Interestingly, the public attitudes towards PWS among populations that share almost the same cultural characteristics, however with higher economic indices, were more positive than the Egyptian sample. Two studies conducted on Arab parents (n = 424) and teachers (n = 202) from Kuwait showed that their public attitude was more positive towards PWS than the Egyptian sample although they reported clear deficits in the knowledge about stuttering [2, 3]. In line with this suggestion, the results of our study revealed 57% higher odds of positive attitudes amongst people with average to high income compared with low income. Still, further research including more detailed questions about the annual income and the occupational status is needed to confirm this finding. On the other hand, a study conducted on different family generations from Turkey, which also has similar cultural and economic characteristics to those of Egypt, showed scores that were very close to the results of this study [29]. One more common criterion between the Egyptian and the Turkish samples is that stuttering was widely considered as God’s will in both societies. Consequently, participants might have been holding a belief that stuttering is a sort of God’s punishment which could partially explain their negative attitudes towards PWS. The association between how religious people are and their attitudes towards stuttering needs further investigation. However, our findings highlight the possible role of the cultural and economic factors in determining the attitudes towards PWS.

Unexpectedly, people having children in this study were less likely to show positive attitudes towards PWS. However, people with children in this study had significantly lower education and income than people with no children. Despite we adjusted our results for education and income, there is a possibility of residual confounding.

In terms of sources of knowledge about stuttering, the Egyptian participants tended to choose TV, radio, and films which coincided with the reference database. Having a common source of knowledge could explain the equal mean ratings for the knowledge component between the Egyptian sample and the reference database. However, the strikingly low score of degree of stuttering knowledge in this study and the reference database should turn a spotlight on the contents of the TV and radio shows that use stuttering and PWS as materials of sarcasm.

Although this study described, for the first time, the public attitudes towards PWS in South Egypt using a multi-stage random sampling approach, some limitations should be considered. First, the sociodemographic characteristics of the participants may not typically resemble that of the general population in Beni-Suef or other cities in South Egypt due to the relatively larger proportion of educated participants in this study. Second, collecting the data by interview rather than self-administration could make the answers vulnerable to socially desirable response bias.

In conclusion, people in South Egypt carry negative beliefs and inadequate knowledge about stuttering and hold insensitive attitudes towards PWS. This emphasizes the need for stuttering education to raise people’s awareness and provide evidence-based information about all aspects of stuttering that hopefully would lead to more empathetic and sensitive interactions with PWS. Future research focusing on the effect of the cultural and economic status in molding such attitudes towards PWS should be considered.

## Supporting information

S1 Data (XLSX)
